# A Reinforcement Learning Based Transmission Parameter Selection and Energy Management for Long Range Internet of Things

**DOI:** 10.3390/s22155662

**Published:** 2022-07-28

**Authors:** Yassine Yazid, Antonio Guerrero-González, Imad Ez-Zazi, Ahmed El Oualkadi, Mounir Arioua

**Affiliations:** 1 Laboratory of Information and Communication Technologies (LabTIC), Ecole Nationale des Sciences Appliquées de Tanger, Abdelmalek Essaadi University, Tangier BP 1818, Morocco; aeloualkadi@uae.ac.ma (A.E.O.); m.arioua@uae.ac.ma (M.A.); 2 Department of Automation, Electrical Engineering and Electronic Technology, Universidad Politécnica de Cartagena, Plaza del Hospital 1, 30202 Cartagena, Spain; antonio.guerrero@upct.es; 3 National School of Applied Sciences of Fez (ENSAF), Sidi Mohamed Ben Abdellah University, Fez BP 2626, Morocco; imad.ezzazi@usmba.ac.ma

**Keywords:** LPWAN, IoT, LoRa, CSS modulation, ADR, MDP, energy efficiency, reliability

## Abstract

Internet of Things (IoT) landscape to cover long-range applications. The LoRa-enabled IoT devices adopt an Adaptive Data Rate-based (ADR) mechanism to assign transmission parameters such as spreading factors, transmission energy, and coding rates. Nevertheless, the energy assessment of these combinations should be considered carefully to select an accurate combination. Accordingly, the computational and transmission energy consumption trade-off should be assessed to guarantee the effectiveness of the physical parameter tuning. This paper provides comprehensive details of LoRa transceiver functioning mechanisms and provides a mathematical model for energy consumption estimation of the end devices EDs. Indeed, in order to select the optimal transmission parameters. We have modeled the LoRa energy optimization and transmission parameter selection problem as a Markov Decision Process (MDP). The dynamic system surveys the environment stats (the residual energy and channel state) and searches for the optimal actions to minimize the long-term average cost at each time slot. The proposed method has been evaluated under different scenarios and then compared to LoRaWAN default ADR in terms of energy efficiency and reliability. The numerical results have shown that our method outperforms the LoRa standard ADR mechanism since it permits the EDs to gain more energy. Besides, it enables the EDs to stand more, consequently performing more transmissions.

## 1. Introduction

The IoT (Internet of Things) and Internet of everything (IoE) technologies are revolutionizing the world into modernity [1]. The connected objects impact the entire world and emphasize new consistent communication aspects that will enable smooth connectivity between objects and humans. The short-range technologies have provided low power and short distance communications as they rely on small-order communication protocols standards. For instance, Bluetooth [2], Wi-Fi, Z-Wave, ZigBee and 6LoWPAN [3,4]. However, these technologies remain constrained as they can not afford long-range connectivity with low energy consumption, which is a tremendous requirement for new IoT applications. Many constraints have been encountered, such as defective efficiency in terms of energy consumption, latency, scalability, and network equipment costs. Afterward, these critical requirements have encouraged the research community to design a new and effective alternative to provide long-range connectivity and low power communications. Consequently, Low Power Wide Area Networks (LPWAN) have been raised as a practical alternative to short-range and low power communications [5]. Besides, LPWAN networks are regarded as a new generation of IoT that relies on new communication techniques to guarantee long transmission ranges and lifespan requirements.

LPWAN technologies have emerged in two categories; cellular and non-cellular [6]. The configuration of these networks is structured to achieve widespread communications and scalable coverage in miscellaneous IoT fields in different environments types and application classes. Within this context, many long-range communication paradigms have been adopted in IoT applications such as Sigfox [7], INGENU [8], Weightless SIG [9], DASH7 [10] and Long Range (LoRa) [11]. However, LoRa has been much attention in IoT field. It operates in the unlicensed industrial-scientific-medical (ISM) frequency bands [12]. The IoT-based LoRa network connects end nodes (EDs) to their gateway (GW) through direct communications. These EDs are typically battery-based. Therefore, it is paramount to accurately assess the IoT-based LoRa system’s energy model. Accordingly, determining the optimal LoRa communication parameters has been challenging for IoT networks.

In LoRa networks, the transmitted information should undergo several processing steps (e.g., whitening, channel encoding, interleaving and modulation). Therefore, the trade-offs between transmission energy and computational energy should be considered to measure the energy efficiency of a typical set of parameter selections. Within this context, many works have addressed the problem of energy modeling and transmission parameter selection in LoRa networks [13,14,15]. However, the proposed models have not considered all the main factors behind the energy cost in LoRaWAN, which can lead to underestimating the lifespan of the EDs, thus the lifespan of the overall network. For instance, the provided energy models in [13,16], have considered only the default available coding rates by LoRa standard. In addition, the authors have adopted relative fixed values for computation energy which is considered impractical as processing energy varies from one transmission setting to another. Furthermore, other works have proposed adaptive algorithms for the LoRa parameter setting [17,18]. However, the related models are based predominately on transmission energy to select an adequate set of parameters. Whereas it is should be more practical to set a coding rate index and then change the spreading factors (SFs) as in some cases; using higher SF with lower coding rate (CR) is better than using lower SF with higher CR. This may lead to gain a considerable amount of energy better than the stand LoRa adaptive data rate (ADR). Moreover, the ideal policy to select the optimal transmission parameters set (e.g., modulation, coding) taking into account the energy-reliability performance has been neglected in most of the recent works. In this paper, we focus on the LoRaWAN protocol elements and the mechanisms of estimating the EDs battery lifetime. LoRaWAN technology uses different default parameters to optimize the energy consumption of the EDs. Therefore, we propose a full energy consumption model for EDs using LoRa modulation and LoRaWAN protocol. The main aim is to use all the possible transmission combinations among SFs and CRs. Besides, investigating the impact of channel medium, SFs, CRs, payload size, and communication range on the energy consumption and reliability of LoRa EDs. In this paper, we have proposed an accurate alternative for default LoRa stand ADR based on Markov Decision Process (MDP) that aims to estimate the optimal transmission parameters to provide increased energy-reliability policies. This scheme is apt to enables significant energy gain, high reliability and adaptive selection of the LoRa transmission parameters regarding the channel condition, the distance, and energy residual of the EDs. The contributions of this work can be summarized as follows:

- We derive a completed LoRa communication system based mainly on the Hamming channel coding scheme and CSS modulation technique. Likewise, thorough details on the functioning and purpose of each system block are deeply detailed. The communication system model includes the channel encoding/decoding, modulation/demodulation, whitening/ de-whitening, and interleaving/de-interleaving processes;
- We provide a full energy model that features the main mechanisms behind energy consumption in the LoRa communication system based on CSS modulation SFs hybrid settings with coded transmissions using different CRs under the Additive White Gaussian Noise (AWGN) communication channel. The energy model addresses the effect of the processing unit, channel coding, and decoding on energy consumption;
- We propose an optimal adaptive algorithm for LoRa parameters selection based on MDP to guarantee energy efficiency and high-reliability trade-offs. The main objective is to find adequate settings that allow LoRa devices to withstand longer, considering the transmission quality and the energy efficiency;

The remainder of the paper is structured as follows. Section 2 presents related works. Section 3 presents a detailed LoRa communication chain. Section 4 provides an investigation of the energy efficiency of LoRa and proposes an energy model for the LoRa communication system. Section 5 details the proposed adaptive parameter selection approach dedicated to LoRa class A EDs. Section 6 discusses the obtained results. Finally, Section 7 provides the conclusion.

## 2. Related Works

Numerous studies have been provided to understand the technical limits of LoRa technology. These works have focused on different layers of LoRa including the application layer [19], transport layer [20], network layer [21], data link layer [22] as well as the physical layer [23]. The first attempts started by investigating the secret of the LoRa modulation technique based on the Chirp Spreading Spectrum (CSS) scheme as reviewed in [24,25]. Moreover, the effect of the modulation chirps’ orthogonality issues has been studied in [26,27,28]. Furthermore, the work [29] has been considered the first work that proposed a primary model based on the LoRa communication chain. This model incorporates mainly the CSS demodulation, de-interleaving, de-whitening, and then channel decoding using a Hamming encoding [30]. Moreover, the authors in [31,32,33] have presented a model that estimates the bit error rate (BER) behavior of LoRa CSS based modulation of diverse SFs under AWGN channel conditions. Besides, to investigate the performance of LoRa under different channel circumstances, authors in [34] have derived a study of LoRa modulation BER under different channels among Nakagami-m, Rayleigh, and Rician fading.

To optimally select the LoRa physical parameters for a standard transmission, authors in [35] have proposed a theoretical ADR control model based on a logistic regression algorithm. In [36], the capture effect was investigated to address the transmit power allocation of poor-conditioned LPWAN EDs among LoRaWAN. The authors of [37] have investigated the ADR technique’s performance and security properties for battery-powered LoRa devices that transmit data on cattle location and health. Another work proposed a new MAC protocol called DG-LoRa and evaluated that varying the number of gateways, channel BW, and CR improves the number of re-transmissions better than the default LoRa method [38]. The impact of the packet size on the LoRa performance was evaluated in [39]. The study aimed to balance the reliability, delay, and energy consumption of LoRa under different physical layer parameters.

Additionally, a platform to assess different stages of LoRa transceivers with a deep emphasis on modulation and demodulation techniques were proposed in [23]. However, this model can only deal with small-sized LoRa packets. On the other side, several works have addressed both the LoRa reliability and energy dissipation requirements. For instance, a first paper addressed the impact of LoRa transmission parameter selection on the performance of LoRa networks, in which the authors studied the impact of LoRa parameters on the system’s energy efficiency and communication reliability through an adaptive method for parameter selection [18]. Moreover, to assess the issue that the nodes near the gateway are more likely to transmit a packet successfully than distant ones, a scheme was introduced to optimize the packet error fairness on LoRaWAN networks [40]. In [41], the authors had studied the effect of SFs allocation of LoRa EDs to optimize the energy consumption constraint using a distributed genetic algorithm and Markov Decision Process respectively. These methods have provided acceptable performance of packet reception probability with a reduced energy consumption amount. Authors in [42,43] have addressed the energy consumption issue in LoRa EDs by proposing a delimitation technique that evaluates the radio propagation behavior of the EDs in the network. The work [44] investigates the optimal SF parameter and transmission power ($P_{tx}^{})$ allocated to EDs in LoRa networks. The other efforts have been dealing with the issue of sensor data collection delay in urban areas focusing on LoRa based gateways [45]. Moreover, the energy consumption and reliability trade-off were also considered in [46]. The authors experienced the energy measurement on different LoRa Semtech EDs, and they have demonstrated the transmission configurations and the channel type impact the energy/reliability trade-off in LoRa networks. In the same context, a prediction model of energy consumption and a probabilistic approach based on Markov’s chain is provided to estimate the lifetime of the LoRa wireless sensor network using the Labview simulation tool [47].

Analyzing these works, we have recognized that the energy efficiency gained by the channel encoding and decoding along with the adequate SF parameter has not been addressed to assess the LoRa energy performance. Besides, the critical energy-reliability tradeoff of LoRa transmission systems has not been addressed in most cases, yet its impact on the energy and reliability performances is of paramount importance. Moreover, the ideal policy to select the optimal set of transmission parameters (e.g., modulation, coding, interleaving and whitening) taking into account the energy-reliability performance has been neglected in most of the recent works. For instance, most of the related works have used the default specified LoRaWAN CR 4/5, the CR 4/7 and occasionally the CR 4/8 along with CSS modulation scheme without considering the channel, residual energy and transmission/processing trade-offs effects. Moreover, we have noticed that the used energy models in previous works are exclusively based on CSS modulation parameters and in few cases on fixed coding, neglecting the interleaving and whitening effects on energy and reliability efficiency on the one hand, and the processing costs of all the transmission blocs on the other hand. These works have been typically based on CSS modulation and demodulation technique according to the channel variation without addressing the channel coding performances in both energy conservation and reliability. Consequently, this work considers a practical and real LoRa transmission scenario by considering the effects of all the transmission blocs and their transmission rates (e.g., CR, SF, BW) in the energy efficiency, reliability and delay performances bearing in mind the energy-efficiency trade-offs resulting in the transmission-processing operations at each transmission round.

To deal with the mentioned gaps in the LoRa communication system, we re-investigated the required transmissions and processing operations and provided a completed and detailed energy-reliability model to practically assess the energy and reliability efficiency gained by each configuration selection. Specifically, we have reconstructed a completed LoRa communication system model that includes the channel encoding/decoding, modulation/demodulation, whitening/de-whitening and interleaving/de-interleaving processes by considering the effects of each transmission selection of all blocks. Within this context, and knowing the effect of each parameter selection on energy and reliability performances, the optimal transmission configuration according to the channel condition, distances and the residual energy of LoRa nodes can be efficiently obtained at each transmission slot. Consequently, based on the provided system model, an optimal adaptive parameter selection scheme is required to meet both reliability and energy efficiency performances. Thus, this work proposes an adaptive LoRa transmission algorithm to provide the optimal policy in terms of transmission parameters selection at each transmission slot, which considers the energy-reliability tradeoffs, distances, channel conditions and residual energy.

## 3. System Model

**Figure 1:**
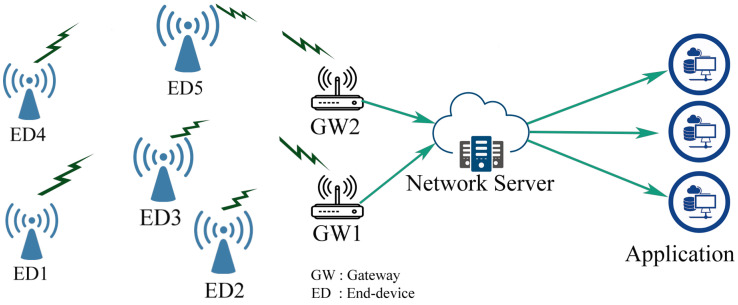
LoRaWAN network architecture.

LoRaWAN is an open standard exhibited by Semtech corporation. It is referred to as an MAC protocol that assure on air wireless inter-connectivity and time scheduling between end nodes and the base station in the network. Additionally, the nodes are densely dispersed, forming a star of star network topology as shown in Figure 1. The GWs communicate the received data to their servers at the back-end. Accordingly, those nodes can be of different classes depending on the requirements of the application: Class A, B, and C [48,49].

Generally, LoRa is referred to as the physical layer basis of the long-range LPWAN technology launched by Semtech. Fundamentally, it uses a proprietary CSS-derived technique by spreading multiple chips over the occupied frequency bandwidth. The basis chirp changes its frequency values instantaneously by wrapping all associated frequency band. For LoRa, the basic chirp is integrated considering 5 main parameters: Bandwidth (BW), SFs, CRs, $P_{tx}^{}$, and channel Signal to Noise Ratio (SNR). The SF, BW and CR can be selected from a set of SF ∈{7,8,9,10,11,12}, BWs ∈{125kHz,250kHz,and500kHz}, and CR ∈{4/5,4/6,4/7,4/8} respectively. The modulator produces different chirps (up-chirps, down-chirps). Concerning the bandwidth, LoRa may transmit a sample every $Tc=\frac{1}{BW}$ which may also denote the chirp duration. Each sample holds a portion of the encoded information to the SF number of bits. These data are encoded again into a non-binary symbol that may take value in {0, 1, 2, *…*, $2^{SF}-1$} before being modulated. Therefore, the symbol is overlaid by changing the chirp signal frequencies over the BW with $2^{SF}$ times $T_{c}^{}$. Thus, a symbol is transmitted every symbol duration $T_{s}^{}=\frac{2^{SF}}{BW}$. Thereby, the higher the SF is, the longer the symbol takes to be transmitted. Additionally, LoRa includes Forward Error Correction (FEC) codes, which can combine blocks of data of four bits each, then encode them by adding bits of adjustable parity from one to four bits, which makes it possible to obtain different coding rates CR ∈{4/5,4/6,4/7,4/8}. Furthermore, the useful bite rate $R_{b}^{}$ is relatively proportional to the used BW, the SF, and the CR, as expressed by:

(1) $$R_{b}^{}=SF\times \frac{BW}{2^{SF}}\times CR.$$

To transmit a packet of several symbols, a combination of up chirps and down chirps are used to refine wireless radio transmissions under allowed ISM free exploitable frequency bands. The LoRa packet generally contains four elements—a preamble, a header, a payload, and a Cyclic Redundancy Check (CRC) block [48]. LoRa relies on the physical layer parameters and possesses specific blocks to convey a packet through large distances. Before any succeeded LoRa transmission, a chain of concatenating processes is performed by the LoRa device transceivers.

Furthermore, LoRa induces controllable redundancy bits into transmitted data to make transmissions resilient to channel medium noises. Accordingly, the data in this method are encoded using a Hamming channel encoding scheme with variable code-words regulated by the parameter CR ∈{4/5,4/6,4/7,4/8}. A data of 4 bits is then encoded cyclically by using the adequate CR to form a code-words of n bits. To improve the reliability, LoRa adopts an extended version of Hamming by using additional CRs. Therefore, the channel encoding generates for each block of four bits a code-word of $\left(4+\alpha_{cr}^{}\right)$ bits, where $\alpha_{cr}^{}\in \left\{1,2,3,4\right\}$.

Additionally, the Hamming check matrix *H* for each code of length and dimension (n,k) is a generator matrix of each orthogonal code *C* denoted by $C_{i}^{T}$. For instance, if *H* is the check matrix for *C*, *H* is an (n−k)×k matrix; the rows are orthogonal to *C* and {x|M×T=0} = *C*. The result from the block of encoded code-words $(4+\alpha_{cr}^{})\times q$ is then reordered to prepare symbol generation using the LoRa spreading factors and add some extra resilience to those code-words by interleaving them. Before that, a whitening block adds known pseudo-random bits to the output of the channel encoded block by operating the XORing process. The length of the whitened vector with the encoded vector must have the same length. Generally, this operation is applied to induce randomness into the symbols to provide more features for clock recovery at the receiver. Afterward, an interleaving operation is performed by taking each queued codeword at the output of the FEC coding block and scattering them over time. This operation serves to separate and reorder the positions of probable errors that may occur. Therefore, channel decoding methods increase the probability of correcting these errors in the receiver decoding processes. Typically, the interleaver shuffles the bits in a representative order to avoid the occurrence of an error, yet it can provide neither error correction nor error detection. Thus, no gain is attached to the processes. Indeed, in LoRa physical layer, this process is utilized mainly to reconstruct the encoded code-words diagonally in the adequate form with the selected spreading factors for the CSS modulation [24].

(2) $$S_{m}^{}=\sum_{p=0}^{SF-1}V_{m}^{}\times 2^{p}.$$

Each vector $V_{m}^{}$ forms symbols of binary digits $S_{m}^{}$ that takes values in {0, 1, 2, 5, *…*, $2^{SF}-1$}. Consequently, the total generated symbols may be surveyed as a vector of *M* elements (i.e., $S_{0⩽m⩽M-1}^{}=\left(S_{0}^{}\;S_{1}^{}\;S_{2}^{}\;S_{3}^{}\;S_{4}^{}\;...S_{M-1}^{}\right)$).

After applying gray indexing, the symbol generation process is issued by translating the interleaved binary code-words of length SF into non-binary symbols. Those symbols may take values in the interval of $\left[0,2^{SF}-1\right]$ possibilities. Thus, each symbol that can be transmitted in a certain defined period also depends on the SF and the chirp duration $T_{c}^{}$. For instance, if an SF of length seven is selected, the value of a symbol may vary from 0 to 255.

Each generated symbol is mapped to the CSS modulator. The modulation procedure relies on specific criteria to give each transmitted packet a standalone form against channel medium interference. Accordingly, each segment of the featured information by non-binary symbols is spread over a time-frequency band using CSS chirps. The CSS modulator relies typically on the on-base chirp waveform, especially to carry each symbol by spreading it over the bandwidth within $T_{s}^{}$ duration. Hence the base-band chirp holding the symbols $S_{0<m<M-1}^{}=0$ is represented in the time domain as:

(3) $$C_{0}^{}\left(t\right)=A\left(t\right)\times e^{\pi j\frac{BW}{T_{s}^{}}t^{2}+\varphi_{0}^{}},$$

where A(t) is the amplitudes that denoted by: $\frac{1}{\sqrt{2^{SF}}}$, *t* is time variate and $\varphi_{0}^{}$ is the initial phase.

Generally, each modulated symbol $S_{0<m<(M-1)}^{}$ is denoted by:

(4) $$C_{S_{m}^{}}^{}\left(t\right)=A\left(t\right)\times e^{2\pi j(\frac{BW}{2T_{s}^{}}t^{2}+\frac{S_{m}^{}}{T_{s}^{}}t+\varphi )}.$$

Assuming the sampling frequency is equal to the bandwidth BW, we obtain the expression of the previous chirping process in the discrete domain due to the Shannon sampling theorem. Consequently, assuming φ is null in the discrete-time domain, the expression of the waveform carrying a single symbol $S_{m}^{}$ within $T_{s}^{}$ duration is:

(5) $$C_{S_{m}^{}}^{}\left(kT_{c}^{}\right)=A\left(kT_{c}^{}\right)\times e^{2\pi j\left(\frac{BW}{2T_{s}^{}}\times kT_{c}^{}+\frac{S_{m}^{}}{T_{s}^{}}\right)\times kT_{c}^{}},\forall k\in Z.$$

Therefore, the spread chirps involving the packet of the encoded symbols are denoted in the discrete domain by:

(6) $$T_{X}^{S_{m}^{}}\left(kT_{c}^{}\right)=\sum_{m=0}^{M-1}C_{S_{m}}\left(k-m\times 2^{SF}\right).$$

Afterwards, the system sends the modulated symbols sequentially over the radio channel. The format of the entire collected message at the receiver input is:

(7) $$R_{X}^{}\left[n\right]=h\times T_{X}^{S_{m}^{}}\left[n\right]+Z\left[n\right],$$

where *h*, $R_{X}^{}\left[n\right]$, $T_{X}^{S_{m}^{}}\left[n\right]$ and *Z*[*n*] are the block-fading channel, the received signal, the transmitted signal and the Zero-mean Gaussian noise with valued variance $\sigma^{2}$ respectively.

The demodulation technique occurs in two distinct phases; the de-chirping and the extraction of the probably received symbols. The de-chirping operation aims to extract the information from the analog received signal by multiplying this signal with the base of the transmitted chirp signal. Regularly, the de-chirping operation extracts out the part containing the transmitted symbols ready to be addressed in the subsequent runs. The resulted signal after applying the de-chirping operation takes the form of an M-ary Frequency Shift Keying (FSK) modulated signal as follows:

(8) $$R_{X}^{d}\left[k\right]=R_{X}^{}\left[k\right]\times e^{-\pi \frac{BW}{T_{s}^{}}\times {\left(kT_{c}^{}\right)}^{2}},\forall k\in Z.\;$$

The LoRa demodulation extracts a set of symbols from the de-chirped signals. Herein, particular coherent demodulation is a crucial strategy to deal with the M-FSK signals after crossing an AWGN channel. Moreover, this modulation technique is more resilient against channel impairment, including the AWGN. Moreover, applying the Discrete Fourier Transform (DFT) enables us to recuperate the transmitted symbols [23].

(9) $${\hat{S}}_{m}^{}=Arg_{max}^{}\left\{Re\left\{DFT\left\{R_{X}^{d}\left(k+m\times 2^{SF}\right)\times S_{m}^{}\right\}\right\}\right\}.$$

The application of the DFT process extracts the recovered non-binary symbols ${\hat{S}}_{m}^{}$. Then, they are coded into binary frames using the gray indexing method, preparing the bit sequence to be declined. The de-whitening processes are applied to withdraw the bit-stream form by re-XORing the binary stream of the received symbols. The expression of de-whitening is given by XORing the demodulated with the same applied whitening sequence of bits. Afterward, an inverse of the interleaving process at the transmission side allows us to store one codeword at a time in the same rectangular array format in a row-wise manner. Furthermore, the vector array of the received bit-stream analysis is the input of the decoding block to check the occurred number of errors. The FEC decoding of the de-interleaved bits relies on the Hamming decoding algorithm. This procedure allows the system to recognize and investigate the LoRa received packet by providing enforcement against the channel to measure the number of occurred bit errors.

## 4. Energy Efficiency of LoRa

A LoRa network incorporates multiple small devices with small batteries, thus their energy is limited. Therefore, the network’s lifespan depends on the adopted protocol to manage the power source of the nodes. Each wireless sensor performs many processes before succeeding a single transmission. Hence, to estimate the overall network energy consumption, a thorough consideration should address all the energy consumption provokers by each single node.

### 4.1. Energy Modeling of LoRa/LoRaWAN

The on-off transmission mechanism is integrated into the node’s hardware system to maintain energy management. This strategy has become increasingly important in improving energy saving for most wireless sensors. Besides, LoRa nodes are activated only for a particular time $T_{active}^{}$ and then remain silent during sleeping mode $T_{sleep}^{}$. In standby mode, the node listens to the radio using clocks that ordinarily do not consume as much energy as during active mode, where it operates several activities that drain the node’s energy supply. Consequently, each packet may last for a total duration of $T_{packet}^{}$ expressed by:

(10) $$T_{packet}^{}=T_{preamble}^{}+T_{payload}^{},$$

where $T_{packet}^{}$, $T_{preamble}^{}$ and $T_{payload}^{}$ are the full packet duration, the preamble duration and the physical payload duration respectively. The preamble duration depends on the used device chip, whereas the payload duration depends on the number of symbols holding the important data [50]. Their values are expressed in function of symbol duration $T_{s}^{}$ by:

(11) $$T_{preamble}^{}=\left(4.25+N_{Pr}^{}\right)\times T_{s}^{}$$

(12) $$T_{payload}^{}=N_{phy}^{}\times T_{s}^{}.$$

The transmission energy in function of transmission power $P_{tx}^{}$ and $T_{packet}^{}$ is expressed by:

(13) $$E_{tx}^{}\left(t\right)=\frac{T_{packet}^{}\times P_{tx}^{}\left(t\right)}{\zeta_{a}^{}},$$

where $\zeta_{a}^{}$ is a fraction related to the node’s antenna amplifier power efficiency. The transmission power $P_{tx}^{}$ typically differs from the uncoded, coded and adopted modulation techniques. Besides, the transmission power for uncoded transmission is expressed by:

(14) $$P_{tx,u}^{}\left(t\right)=\eta_{}^{}\frac{E_{b}^{}}{N_{0}^{}}\times N\times L_{p}^{},$$

where $\eta_{}^{}$, $E_{b}^{}$, $N_{0}^{}$ are the system’s spectral efficiency, the required minimum energy per bit at the receiver side, and the noise power spectral density respectively. $L_{p}^{}$ is the path loss estimation which is defined by:

(15) $$L_{p}^{}={\left(\frac{4\pi f_{c}^{}}{c}\right)}^{2}\times {\left(\frac{d}{d_{0}^{}}\right)}^{\alpha }+\chi$$

(16) $$N=T\times K\times BW\times 10^{\left(\frac{N_{F}^{}}{10}\right)},$$

where $f_{c}^{}$, *c*, *d*, $d_{0}^{}$, $N_{F}^{}$, and χ are: the carrier frequency, celerity, distance between transmitter node, the GW and $d_{0}^{}$ initial distance which is fixed in this study to one meter, noise figure and α is the path loss exponent which depends on the environment characteristics respectively and the standard deviation used only when there is a shadowing effect.

Besides, the transmission power for non coded packets is given by:

(17) $$P_{tx,u}^{\left(t\right)}=\eta_{}^{}\times 10^{\left(\frac{\gamma_{u}^{}+N_{F}^{}}{10}\right)}\times T\times K\times BW\times L_{p}^{},$$

where $\gamma_{u}^{}$ is the required SNR for non-coded transmissions, λ is the transmitted wavelength corresponding to $f_{c}^{}$, *K* is the Boltzmann constant, *T* is the absolute temperature in Kelvin and BW is the bandwidth.

Given the SNR gain by using an FEC code, the required power for encoded transmission can be expressed in function of $P_{tx,u}^{}$ and coding gain $G_{c}^{}$ as follows:

(18) $$P_{tx,c}^{\left(t\right)}=\frac{P_{tx,u}^{\left(t\right)}}{10^{\left(\frac{G_{c}^{}}{10}\right)}}.$$

The utilization of LoRa modulation based on the CSS scheme enables the system to perform transmission in noisy channel mediums. The CSS modulation scheme provides crucial energy savings as LoRa uses different SFs, resulting in different gains. We rely on the gain measures expression below for different SFs. We define SNR gain as the additional SNR in dB required by CSS modulation schemes that allows the SFs to achieve the same BER performance target. Hence, SNR gain for a given target BER for coded $G_{c,css}^{}\left(SF\right)$ transmitted packets can be expressed $\forall \;SF_{i}^{}\neq SF_{j}^{}\in \left\{7,8,9,10,11,12\right\}$ by:

(19) $$G_{c,css}^{}\left(SF_{i,j}^{}\right)=\gamma_{c,css}^{}\left(SF_{i}^{}\right)-\gamma_{c,css}^{}\left(SF_{j}^{}\right).$$

Thus, to measure the minimum required power between two separate spreading factors $SF_{i}^{}$ and $SF_{j}^{}$; the following expression can be used:

(20) $$P_{tx,css}^{\left(t\right)}\left(SF_{i}^{}\right)=\frac{P_{tx_{css}^{}}^{\left(t\right)}\left(SF_{j}^{}\right)}{10^{\left(\frac{G_{css}^{}\left(SFi,j\right)}{10}\right)}}.$$

Typically, any LoRa ED spends its energy cooperatively by performing several tasks. In general, the node may take two states; active and sleep state. A significant amount of energy is consumed in active mode than in sleep mode. The total energy consumed per node taking into account both states is expressed by:

(21) $$E_{Tot}^{\left(t\right)}\left(ED_{id}^{}\right)=E_{sleep}^{\left(t\right)}\left(ED_{id}^{}\right)+E_{act}^{\left(t\right)}\left(ED_{id}^{}\right),$$

where $E_{sleep}^{\left(t\right)}$ and $E_{act}^{\left(t\right)}$ are the total consumed energy during sleep and active mode respectively. The energy consumed in sleep mode is proportional to $P_{sleep}^{\left(t\right)}$ within sleep duration $T_{sleep}^{\left(t\right)}$ by:

(22) $$E_{sleep}^{\left(t\right)}\left(ED_{id}^{}\right)=P_{sleep}^{\left(t\right)}\times T_{sleep}^{\left(t\right)}.$$

In active mode, the node is required to perform multiple principal operations which leads to drain energy such as Radio Frequency (RF), CPU, and then circuity-sensing energy that englobes both consumed energy for sensing and circuit tasks.

(23) $$E_{act}^{\left(t\right)}\left(ED_{id}^{}\right)=E_{rf}^{\left(t\right)}+E_{proc}^{\left(t\right)}+E_{cs}^{\left(t\right)}.$$

#### 4.1.1. Radio Communication Energy

The communication module includes both up-link transmission and down-link receptions. The RF is responsible for end-nodes data transmission and reception. Typically, to transmit a packet, the node starts by sending two up-link notifications in a specific time duration, then it prepares to receive the feedback by opening sequentially two listening windows. If there is an absence of detection during the first opening, the node opens a second window. If no reception occurs during both windows opening, the node will wait for the next duty cycle to re-transmit the message until the link exchange succeeds. At the emitter node, after the expiry of the allowed number of up-link trials within $T_{preamble}^{}$, it initiates the first listening window during $T_{w1}^{}$, if nothing is detected, it tries again by opening a second window of $T_{w2}^{}$ duration, otherwise it transmits the same preamble waiting for the next duty cycle.

The energy consumption during this radio communication processes is estimated by the following expression:

(24) $$E_{rf}^{\left(t\right)}\left(ED_{id}^{}\right)=E_{rx}^{\left(t\right)}\left(ED_{id}^{}\right)+E_{tx}^{\left(t\right)}\left(ED_{id}^{}\right),$$

where $E_{rx}^{\left(t\right)}\left(ED_{id}^{}\right)$ is the energy consumed during reception period and $E_{tx}^{\left(t\right)}\left(ED_{id}^{}\right)$ is the energy consumed during transmission period.

(25) $$E_{rx}^{\left(t\right)}\left(ED_{id}^{}\right)=E_{rx,w1}^{\left(t\right)}\left(ED_{id}^{}\right)+E_{rx,w2}^{\left(t\right)}\left(ED_{id}^{}\right),$$

where $E_{rx,w1}^{\left(t\right)}\left(ED_{id}^{}\right)$ and $E_{rx,w2}^{\left(t\right)}\left(ED_{id}^{}\right)$ are the energies consumed during listening on first window opening and second window respectively.

(26) $$\begin{array}{cc} E_{rx,w1}^{\left(t\right)}\left(ED_{id}^{}\right) & =P_{rx,w1}^{\left(t\right)}\times N_{sym}^{}\left(ED_{id}^{}\right)\times T_{s}^{}, \end{array}$$

where $N_{sym}^{}$ corresponds to the number of symbols associated with up-link and down-link communications and its value depends on the selected SF. $P_{rx,w1}^{\left(t\right)}$ is the needed transmission power during $T_{w1}^{}$, and $T_{s}^{}$ is the symbol duration.

(27) $$\begin{array}{cc} E_{rx,w2}^{\left(t\right)}\left(ED_{id}^{}\right) & =P_{rx,w2}^{\left(t\right)}\left(ED_{id}^{}\right)\times \left(\frac{2^{SF}+32}{BW}\right), \end{array}$$

where $P_{rx,w1}^{\left(t\right)}$ is the required transmission power during $T_{w2}^{}$, and BW is the bandwidth.

#### 4.1.2. Computation Energy

In addition to the energy dissipated by radio components, the ED’s processing unit consumes energy while treating the data through different sequences; channel coding, whitening, interleaving, gray indexing, and mostly when modulating the signal. The ED consumes energy through switching sequences and leakage current. Thus, in our model, we consider the total time taken by the processed ED’s processing unit to complete packet post-processing as follows:

(28) $$T_{}^{f_{mcu}^{}}=T_{sc}^{}+T_{cc}^{}+T_{whi}^{}+T_{int}^{}+T_{gr}^{}+T_{css}^{},$$

where the time required by the physical block source coding, channel coding, whitening, interleaving, gray conversion and the CSS modulation block are denoted respectively by: $T_{sc}^{}$, $T_{cc}^{}$, $T_{whi}^{}$, $T_{int}^{}$, $T_{gr}^{}$ and $T_{css}^{}$. The energy dissipated by a single node upon the activation state i.e the energy consumed by the processing unite $E_{pros}^{\left(t\right)}$ can be expressed by:

(29) $$\begin{array}{cc} E_{pros}^{\left(t\right)} & =P_{mcu,on}^{\left(t\right)}\times \Phi_{mcu}^{\left(t\right)}+P_{mcu,off}^{\left(t\right)}\times \left(T_{bo}^{}-\Phi_{mcu}^{\left(t\right)}\right)+E_{leakage}^{\left(t\right)}, \end{array}$$

where $E_{leakage}^{\left(t\right)}$ and $\Phi_{mcu}^{\left(t\right)}$ are the number of clock cycles per task. $P_{mcu,on}^{\left(t\right)}$ and $P_{mcu,off}^{\left(t\right)}$ are the consumed power when the CPU is on and off modes, respectively. $T_{bo}^{}$ is the back off time.

(30) $$\begin{array}{c} \begin{array}{cc} E_{leakage}^{\left(t\right)} & =L_{packet}^{}\times V_{dd}^{}\times I_{leakage}^{}\times \left(\frac{\Phi_{mcu}^{\left(t\right)}}{f_{ED_{id}^{}}^{}}\right). \end{array} \end{array}$$

Additionally:

(31) $$\begin{array}{cc} \Phi_{mcu}^{\left(t\right)}= & T_{bo}^{}(\left(1-p_{\alpha }^{}\right)\left(1-p_{\beta }^{}\right)\left(1-p_{0}^{}\right)\left(\tau L+\left(1-p_{f}^{}\right)L_{ack}^{}\right)\\ \; & +\lambda_{s}^{}\times T_{m}^{}+T_{MCU}^{f_{mcu}^{}}). \end{array}$$

Given that, $p_{\alpha }^{}$, $p_{\beta }^{}$, $p_{0}^{}$, $p_{f}^{}$, τ, *L*, $L_{ack}^{}$, $T_{bo}^{}$ and $T_{MCU}^{f_{mcu}^{}}$ denote respectively; the busy channel probability during the first and second window opening, the probability when the gateway queue is empty, probability of fail, the channel access probability, the length of data frame, the length of checking acknowledgment, the back-off time and the processing duration required by the device micro-controller within frequency $f_{mcu}^{}$ and $\lambda_{s}^{}$ is the sensing frequency of the LoRa end-device.

Considering the channel encoding scheme is based on Galois field $GF(2^{m})$, the time required to encode a code sequence of *n* bits operation within a time period $t_{cycle}^{}$ using Hamming code [51,52] can be expressed by:

(32) $$T_{cc}^{}=\frac{n\times (2k-1)}{k}\times t_{cycles}^{},$$

where $t_{cycle}^{}$ is the time required to complete each single coding sequence. Therefore, the processing duration needed to modulate $N_{symbols}^{}$ number of symbols by CSS modulation can be given as:

(33) $$T_{css}^{}=\frac{N_{symbols}^{}}{M_{speed}^{}},$$

where the modulation speed $M_{speed}^{}$ can be expressed by:

(34) $$M_{speed}^{}=\frac{BW}{2^{SF}}\times CR.$$

In this work, the total time required for interleaving, source coding, gray mapping, and whitening is assumed to be negligible as they are practically small compared to other processing operations. Besides, the value of $\Phi_{mcu}^{\left(t\right)}\approx T_{MCU}^{f_{mcu}^{}}$.

#### 4.1.3. Sensing and Circuity Energy

The sensor unit consumes the energy for data acquisition, and digital-analog converters which is estimated by:

(35) $$E_{sc}^{\left(t\right)}\left(ED_{id}^{}\right)=E_{c}^{\left(t\right)}+E_{s}^{\left(t\right)}.$$

The energy dissipated by the node circuitry $E_{c}^{\left(t\right)}$ and sensing unit $E_{s}^{\left(t\right)}$.

(36) $$E_{c}^{\left(t\right)}\left(ED_{id}^{}\right)=E_{idl}^{\left(t\right)}+E_{syn}^{\left(t\right)}+E_{on}^{\left(t\right)}+E_{off}^{\left(t\right)}+E_{led}^{\left(t\right)}+E_{brd}^{\left(t\right)}.$$

The expressions $E_{idl}^{}$, $E_{syn}^{}$, $E_{on}^{}$, $E_{off}^{}$, $E_{led}^{}$ and $E_{brd}^{}$ present respectively the energy dissipated by each single end node in mode: idle lightening, synthesizer to transmission, switching off to on, switching on to off, energy consumed by node Light Emitting Diodes (LEDs) and the beard energy.

(37) $$E_{S}^{}\left(ED_{id}^{}\right)=T_{bo}^{}\lambda_{s}^{}\left(\sum_{i=1}^{N_{s}^{}}P_{s}^{on}T_{m}^{}+P_{adc}^{on}T_{m}^{}+P_{adc}^{off}\left(\frac{1}{\lambda_{s}^{}}-T_{m}^{}\right)+\sum_{i=1}^{N_{s}^{}}P_{s}^{off}\left(\frac{1}{\lambda_{s}^{}}-T_{m}^{}\right)\right),$$

where the time required for total sensing by a node is $T_{m}^{}$, the sensing power in on active mode $P_{s}^{on}$ and off mode $P_{s}^{off}$. Back off duration $T_{bo}^{}$ and $\lambda_{s}^{}$ refers to node’s sensing frequency, consumed power in by ADC activity when the node is active $P_{adc}^{on}$ and $P_{adc}^{off}$ in sleep mode.

### 4.2. Total Energy Dissipation of the Overall Network

The total energy dissipated in the network that comprise $N_{nodes}^{}$ number of LoRa end nodes $ED_{id}^{}$ at time *t* after the occurrence of total number of duty cycles $N_{c}^{}$, is the sum of the total energy dissipated by each ED:

(38) $$E_{network}^{}=\sum_{id=1}^{N_{nodes}^{}}N_{c}^{}\times E_{Tot}^{\left(t\right)}\left(ED_{id}^{}\right).$$

Furthermore, we can also use the following formula to predict the residual energy at specific time in a given ED equipped with initial power capacity $E_{init}^{}\left(ED_{id}^{}\right)$ in function of the number of the occurred transmission cycles.

(39) $$E_{Res}^{\left(t\right)}\left(ED_{id}^{}\right)=E_{init}^{}\left(ED_{id}^{}\right)-N_{c}^{}\times E_{Tot}^{\left(t\right)}\left(ED_{id}^{}\right).$$

## 5. Proposed Method

We assume an extensive LoRa network composed of various autonomous battery-powered LoRa EDs scattered randomly around their GW. Each ED is supposed to convey packets respecting the duty cycle (DC) regulation with minimum energy usage. Thus, the transmission parameters should be adopted carefully to ensure the energy efficiency and reliability trade-off. In other words, if multiple choices are available, the ED has to select the setting that meets the task with the lowest power requirement with high reliability. The GW can successfully decode the received packets if the SNR is above the threshold $SNR_{th}^{}$ for a given selection, and the received signal should necessarily exceed the sensitivity of the receiver antenna $S_{rx}^{}$. If these conditions are not satisfied, the uplink and downlink messages cannot be received. Therefore, the ED re-transmits the packets within a certain number of allowed uplink attempts. The sensitivity of the GW receiver [53] is given as:

(40) $$S_{rx}^{}=-174+10log_{10}^{}\left(BW\right)+N_{F}^{}+SNR_{th}^{}.$$

This work proposes a method that overlaps the energy efficiency and transmission reliability of the LoRa ED. Due to their gains, the study considers both CSS modulation and coding rate energy saving. The EDs power consumption is potentially related to the transmission and surrounding environmental conditions. Therefore, the parameter selection procedure for the physical layer must be handled carefully, respecting the several criteria among transmission parameter adaptation, transmission power, and critical distance $d_{c}^{}$ to maintain energy efficiency and consistency for any ED. Each ED in the network should choose the adequate transmission parameter considering the channel medium variances. As a result, the selected option should prove successful transmissions with low power consumption.

Besides, to remedy the issue of many transmission parameter options availability, to choose the most efficient, a parameter called distance threshold $d_{c}^{}$ was introduced. This parameter is defined as the distance at which any node located far from the GW can successfully convey a typical transmission. The system adjusts the settings until obtaining the highest distance with suitable parameters. Correspondingly, the selection should consider the packet size, processing energy, and the distance that separates any ED from its GW. To estimate this distance, we rely on the following expression:

(41) $$d_{c}^{}={\left(\frac{\left(E_{tx,SFi}^{}-\chi \right)\times 10^{\left(\frac{-\gamma_{css}^{\left(t\right)}\left(SF_{j}^{}\right)-N_{F}^{}}{10}\right)}}{T_{packet(SF_{j}^{})}^{}\times \eta \times T\times K\times BW}{\left(\frac{\lambda }{4\pi }\right)}^{2}\right)}^{\frac{1}{\alpha }}.$$

Any ED in the network must decide which parameter combination is selected to maintain the energy-draining for a given duty cycle. Typically, selecting a single parameter among SF or CR regarding data packet size, the system must settle the parameters continuously, taking into account the critical distance.

Therefore, the main aim to measure the difference of the energy consumed for different SFs, which is, in other words, the gain of energy obtained using two separate SFs. Thus, the energy difference between two consecutive SFs; $SF_{i}^{}$ and $SF_{j}^{}$, i,j∈{6,7,8,9,10,11,12}, and ∀i≠j, is denoted by:

(42) $$\Delta E_{tx}^{}\left(ED_{id}^{}\right)=E_{tx,css(SF_{i}^{})}^{}\left(ED_{id}^{}\right)-E_{tx,css(SF_{j}^{})}^{}\left(ED_{id}^{}\right).$$

Besides, this equation is also applied to get the average consumed energy for processing comparing between two different SF parameters which can be expressed by:

(43) $$\Delta E_{proc}^{}\left(ED_{id}^{}\right)=E_{proc,css(SF_{i}^{})}^{}\left(ED_{id}^{}\right)-E_{proc,css(SF_{j}^{})}^{}\left(ED_{id}^{}\right).$$

The energy consumption of a given set of configurations among SF, CR, BW, and transmission power $P_{tx}^{}$ is considered taking into account the trade-off between the required energy for transmissions and the computation processes. More specifically, for a given transmission scenario, an ED may successfully transmit a packet by using multiple possible settings. Therein, the transmission setting with less energy consumption is more likely to be selected. This may be achieved by assessing the energy efficiency esteemed by the margin difference between $E_{tx}^{}$ and $E_{proc}^{}$ for each possible setting.

This classification is obtained by comparing the total $E_{tx}^{}$ and $E_{proc}^{}$ per cycle for two successive configurations using different SFs with a given $CR_{}^{}$ where SF∈{7,8,9,10,11,12} and CR∈{4/5,4/6,4/7,4/8}. Therefore, the energy saving is defined as the difference of transmission and processing energy consumption between two different settings, and it is expressed as:

(44) $$\Delta E_{}^{}\left(ED_{id}^{}\right)=\Delta E_{tx}^{}\left(ED_{id}^{}\right)+\Delta E_{proc}^{}\left(ED_{id}^{}\right).$$

This expression allows us to investigate the energy performance between various transmission parameters, therefore calibrating the energy differences in terms of energy gain and cost. Moreover, to decide if the selected configuration is relevant in reliability and energy efficiency, we compare multiple settings that execute the same task for the same scenario conditions. Besides, we investigate the energy efficiency ratio that combines both required transmission and computation energy. However, the difference value between two settings $\Delta E_{tx}^{}$ by a given end device $ED{}_{id}$ is considerable if the overall transmission energy is strictly higher than energy processing [54]. Therefore, the energy efficiency metric is expressed as:

(45) $$Ee_{}^{}\left(ED{}_{i}\right)=\frac{\Delta E(ED_{id}^{})}{E_{Tot}^{}}\times 100.$$

Additionally, the reliability using a given set of transmission can be assessed between two different settings to transmit the same packet by dividing the total transmitted packet $L_{packet}^{}$ by the total consumed energy per cycle:

(46) $$\hat{E}e_{}^{}\left(ED{}_{i}\right)=\frac{L_{packet}^{}}{E_{Tot}^{}}\left[bit/J\right].$$

We investigate the system performance in AWGN canal using our redeveloped LoRa communication chain that includes all the previous mentioned physical layer processes. The BER $e_{ber}^{}$ and the packet error rate (PER ) of a coded packet given that the SNR and SF can be derived as follows [55]:

(47) $$PER\left(ED{}_{id}\right)=1-{\left(1-BER\right)}^{\frac{4}{5}\left(l_{packet}^{}-l_{header}^{}\right)}.$$

According to [56], the BER expression of CSS modulation in AWGN channel can be approximated by:

(48) $$BER\left(ED{}_{id}\right)\approx \frac{1}{2}Q\left(1.28\sqrt{\frac{E{}_{s}}{N{}_{0}\times 2^{SF}}}-1.28\sqrt{SF}+0.4\right),$$

where $E_{s}^{}$ depicts the symbol energy, $N_{0}^{}$ is the single-sided noise power spectral density, $Q_{}^{}$ is the distribution function, and $l_{header}^{}$ is the header length. Moreover, the required energy to transmit a coded bit $E_{b}^{tx,c}$ is subtracted from the energy needed to transmit an uncoded bit $E_{b}^{tx,u}$. It can be analytically provided by:

(49) $$E_{b}^{tx,c}=\frac{P{}_{tx,c}}{R_{b}^{}}=\frac{E_{b}^{tx,u}}{10^{\frac{G{}_{c}}{10}}}.$$

Furthermore, the energy consumption can be analytically expressed independently to the data throughput for a typical selected physical parameter. Therefore, the amount of saved energy $\Delta E_{tx}^{}\left(ED_{id}^{}\right)$ is expressed as: $N_{0}^{}$

(50) $$\Delta E_{tx}^{}\left(ED_{id}^{}\right)=E_{b}^{tx,u}\left(1-10^{-\frac{G{}_{c}}{10}}\right).$$

**Figure 2:**
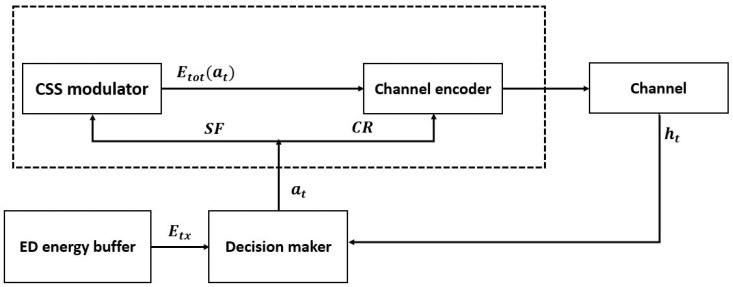
Flowchart of the proposed model.

We adopt a multi-objective optimization method to formulate the LoRa transmissions and processing parameter selection as a Markov Decision Process. The designed optimization model under uncertain surroundings for decision-making is known as MDP. This method is based mainly on the MDP [57] technique to assign the transmission parameters autonomously. The flowchart presented by Figure 2 illustrates the whole idea of the proposed method. Therefore, the main aim is to assign the most energy-efficient configuration to an ED while transmitting a given packet from a given distance to the GW. To assign a given transmission parameter, the ED has to consider many variances affecting transmission quality, such as random channel conditions. The ED should efficiently adjust its transmission parameters by using minimum energy dispenses. At each decision instant, the system is maintained in a particular state S, and the agent elects an action A, existing in the latter state. Once the action A is performed, an intermediate cost C is received by the agent, and thus the system is moved to a qualitatively different new state $S^{\prime }$ based on the transition probability $P_{r}^{}\left(S,A,S^{\prime }\right)$. The main aim of the MDP is to find the optimal policy that minimizes the long term average cost (i.e., $E_{tot}^{}\left(ED\right)$). An MDP can be either a finite or infinite time frame [58]. Given a finite MDP time horizon, the optimal policy $\pi_{}^{*}$ to minimize the total expected cost is defined as follows:

(51) $$min\;V_{\pi }^{}\left(S\right)=F_{\pi ,s}^{}\sum_{t=1}^{T}\gamma_{}^{t}R\left(s_{}^{{}^{\prime }}|s_{}^{},\pi \left(a_{}^{}\right)\right).$$

Here, γ is the discounting factor and F[.] is the expectation function. Furthermore, finite time horizon Markov decision processes are a finite time horizon MDP solution. The system’s performance takes place in a time known as the lifetime measure of the ED. In particular, the system starts in state S or the initial state of the node when it is full of energy and continues to operate until the battery is completely discharged. The optimal policy is to minimize $V_{\pi }^{}\left(S\right)$. If we denote $V_{t}^{*}\left(S\right)$ as the minimum achievable reward at state s, then we can estimate the value of $V_{t}^{*}\left(S\right)$ at each state recursively by solving the following optimal Bellman equations:

(52) $$V_{t}^{*}\left(S\right)=min_{a\in A}^{}\left[R_{t}^{}\left(s,a\right)+\sum_{s^{{}^{\prime }}\in S}^{T}P\left(s_{}^{{}^{\prime }}|s_{}^{},a\right)V_{t+1}^{*}\left(s_{}^{{}^{\prime }}\right)\right]$$

(53) $$Q_{t}^{*}\left(s,a\right)=C_{}^{}\left(s,a,s_{}^{{}^{\prime }}\right)+\sum_{s^{{}^{\prime }}}^{T}P\left(s,a,s_{}^{{}^{\prime }}\right)V_{t-1}^{*}\left(s_{}^{{}^{\prime }}\right).$$

Wherein $V_{t}^{*}\left(S\right)$ is the value of state s and $Q_{t}^{*}\left(S,A\right)$ is the value of taking action A in state S.

The proposed MDP model is defined by the tuple (S, A, $P_{r}^{}$, C(.)), where S presents the state of the system, A is the set of possible actions, $P_{r}^{}$ is the set of transition probabilities of the system states, and C(.) is the cost function for a given decision. We specify each element of the tuple as follows:

- States: There are two different states associated with every ED on the network; a sleep state $S_{sleep}^{}$ and active state $S_{active}^{}$. During $S_{sleep}^{}$ the ED may perform data gathering from its environment and processing tasks but no transmissions can be held, whereas during $S_{active}^{}$, it communicates with the GW base and opens predominately listening windows to send and receive exchanged complete duplex transmissions with the same GW. In an active state, the ED takes many autonomous decisions to adjust transmission settings, including transmission power $P_{tx}^{}$ adjustment. Each state s∈S consists of two components s=[b,h], where S=B×H. B denotes the energy state space, and H denotes the channel medium state space. b∈B is the energy state, B={0,1,…,B} contains all possible energy buffer levels regarding the different possible transitions. The channel medium state h∈H={1,…,H} influences the packet reception probability at the receiver ED and, since we assume a power-controlled transmission system, it also affects the power consumption at the transmitter. The process is used to derive the channel state transition matrix for an AWGN channel model as described in [59,60].
- Action: At each decision epoch *t*, the transmitter ED obeys the system state $s=s_{t}^{}$ and determines an action $a=a_{t}^{}$ from the action set $A_{s}^{}$. In our model $a=a_{t}^{}$ corresponds to the efficient setting SF and CR to be used in the current slot *t*. It compares the energy efficiency of each possible transmission set used by the transmitter in the current time slot *t*. After comparing each possible transition, the ED selects the adequate SF and CR as actions. The ED is assumed to check and test all the possibilities of transmission settings in order to find the optimal policy. This way ensures successful transmissions with high bite rates. The transition to the active state is proportional to the action A(SF,CR) that is handled consecutively by the established transmission parameters and transition probability;
- Transition probabilities: Let *t* and $s=s_{t}^{}\left[b_{t}^{},h_{t}^{}\right]$ express the current time index and the approach state in slot t, respectively. We indicate with at the action taken in the present slot t, and we refer to $E_{tot}^{}\left(s_{t}^{},a_{t}^{}\right)$ as the total consumed energy given that action $a_{t}^{}$ is chosen. Hence, the energy evolves as:
  The transition from the state S to another state $S^{\prime }$ by the action A relies on the efficiency of different possible transmission settings. The selection of an action A is proportional to the transition probability from sleep to active mode. The transition probability from state $s_{t+1}^{}=\left[b_{t+1}^{},h_{t+1}^{}\right]$ given that the action $a_{t}^{}$ is selected is:
  (54) $$b_{t+1}^{}=max\left\{0,minb_{t}^{}-E_{tot}^{}\left(s_{t}^{},a_{t}^{}\right),B\right\}.$$
  If $s_{t}^{}$ is the system state, action $a_{t}^{}$ is admissible only if $E_{tot}^{}\left(s_{t}^{},a_{t}^{}\right)\leq b_{t}^{}$. Additionally, $E_{tot}^{}\left(s_{t}^{},a_{t}^{}\right)$ is given by the sum of two segments: the energy consumption associated with processing $E_{pros}^{}\left(s_{t}^{},a_{t}^{}\right)$ and that associated with the transmission task $E_{tx}^{}\left(s_{t}^{},a_{t}^{}\right)$.
  (55) $$P_{r}^{}\left(s_{t+1}^{}|s_{t}^{},a_{t}^{}\right)=\theta \left(b_{t+1}^{}-b_{t}^{}+E_{tot}^{}\right)\times p_{h}^{}\left(h_{t+1}^{}|h_{t}^{}\right),$$
  where θ(.) refers to the indicator function that is equal to 1 when the argument is zero and zero otherwise. $p_{h}^{}$(.) is the transition probability matrices of the channel.
- Cost function: It is a function that minimizes the total energy that encompasses the transmission and processing energy as defined by Equations (13) and (29). The cost function implicitly selects the adequate transmission parameter that minimizes the assessed energy $E_{Tot}^{}$ per transmission using optimal SF and CR. $P{}_{r}$(.) is the probability that the ED stays in the active mode. The transition from *s* to $s^{\prime }$ by the action *a* is established through the probability of $P{}_{r}$(.) with the cost C(.):
  (56) $$C_{}^{}\left(s,a,s^{\prime }\right)=minE_{Tot}^{}\left(s,a,s^{\prime }\right),$$
  where $E_{Tot}^{}\left(s,a,s^{\prime }\right)$ is the consumed energy by the ED at the current state using optimal selected transition parameters.
  Policy π describes a sequence of decision rules that associate the system state with the action to be taken. The purpose of this policy is to minimize the long-run average distortion, i.e., the long-run average cost. It is driven by the chosen eligible policy π (that decides on the action a).
  (57) $$J_{}^{\pi }=\lim_{N\to +\infty }\frac{1}{N}E_{s}^{}\sum_{n=0}^{N}C\left(s_{n}^{},a_{n}^{}\right).$$
  The main goal of this expression is to determine $J_{}^{☆}=min_{\pi \in \phi }^{}J_{}^{\pi }$ and the corresponding optimal policy $\pi_{}^{*}=argmin_{\pi \in \phi }^{}J_{}^{\pi }$.
- Solution of the MDP: The proposed MDP scheme can be solved via the Value-Iteration Algorithm (VIA), which satisfies the optimal Bellman’s equation. This method is used mainly for infinite-horizon average cost MDPs as in our current study. Generally, VIA defines two parts *J* and *Q*, that are iteratively updated beginning from an initial assessment $J_{0}^{}\left(.\right)$, e.g., $J_{0}^{}\left(s\right)$ = 0, ∀s∈S. Notably, the *t*th iteration determines:
  (58) $$Q_{t}^{}\left(s,a\right)=C_{}^{}\left(s,a\right)+\sum_{s^{{}^{\prime }}}^{T}Pr\left(s,a,s_{}^{{}^{\prime }}\right)J_{t-1}^{}\left(s_{}^{{}^{\prime }}\right).$$
  The long term average cost is expressed as:
  (59) $$J_{t}^{}\left(s\right)=\min\;Q_{t}^{}\left(s,a\right),$$
  where $J_{t}^{}\left(S\right)$ is the value of state *s* and $Q_{t}^{*}\left(s,a\right)$ is the value of taking action A in state S.
  The immediate cost C(s,a) derived in current state s is summed with the expected optimal cost retrieved from the upcoming slot, weighed according to the system dynamics. The span gives the convergence criterion semi-normal operator sp(j)≜min(j)−max(j) characteristic computed for $j=J_{t+1}^{}\left(S\right)-J_{t}^{}\left(S\right)$ the semi-normal of the span guarantees that (59) is a compaction mapping, and thus the algorithm is guaranteed to converge. The iterative algorithm is stopped when sp(.)≤ϵ, at a chosen tolerance threshold ϵ. Consequently, the optimal policy $\pi_{}^{*}$ is then determined by computing the optimal action to take in each state s∈S, i.e., $a_{}^{*}\left(s\right)=Argmin_{a\;inA_{s}^{}}^{}\in Q_{n}^{}\left(s,a\right)$, where *n* is the last iteration of VIA, and has the next key characteristic.

## 6. Results and Discussions

In this section, we compare the performance of LoRa default ADR with our proposed model. We implemented large-scale LoRaWAN simulation tests using the Matlab tool. The simulation environment imitates the real-world behavior of LoRaWAN taking into account all the elements of the transceiver communication chain described in section III. As a channel medium, we considered the AWGN channel. Table 1 shows the parameter settings we used in the simulation experiments. We assumed that payload is variable for the general comparison scenario but fixed at PL = 30 Bytes a specific distance for comparison reason between LoRa default ADR and the proposed method. The class A EDs are randomly scattered within the range of 12 km around a GW.

We limit the transmission power to 20 dBm, the noise figure to 10 dBi, and the path loss to 3. The duty cycle is limited to 1%. In LoRaWAN’s essential operation, multiple channels and bandwidths can be employed. However, for simplicity, only 125 kHz comprehensive single-channel communication is tested. All the activities by the processing units are performed with a 4 Mhz processing frequency. For the energy consumption modeling, we assumed that the EDs consume the energy of 0.25 mJ every detection cycle. The currents $I_{leakage}^{}$, $I_{rx}^{}$ and $I_{sleep}^{}$ are fixed as described in LoRa sensors datasheet [61,62]. The processing and transmission voltages are 2 V and 3.3 V, respectively. Besides, to represent the errors that occurred during transmitting different packet sizes within the AWGN channel medium. The communication chain explained earlier has been explored to tune the appropriate SNR value (i.e., $\gamma_{c,CSS(SF)}^{}$) for BER for coded transmissions using Hamming coding scheme. A packet of random stream bits (e.g., 0 and 1) has been transmitted through an AWGN channel and then decoded for each iteration. Exploring the mentioned blocks at the receiver and the results found in our previous work [32], we assume the SNR versus BER values of each SF at BER = $10^{-4}$ for simplification of calculation. These values have been used to estimate the consumed energy of each transmission configuration of a given ED in the following sections.

### 6.1. Energy and Reliability Evaluation of LoRaWAN

In this subsection, we evaluate the energy and the reliability of LoRa default ADR under different scenarios. The evaluation aims to understand the distribution of the energy consumption of the EDs on the network versus the covered distances using different transmission setting. The main goal of this section is to demonstrate that the LoRa standard ADR is not energy efficient under certain scenarios, therefore considering the transmission and processing energy for parameter selection could provide better performances.

**Figure 3:**
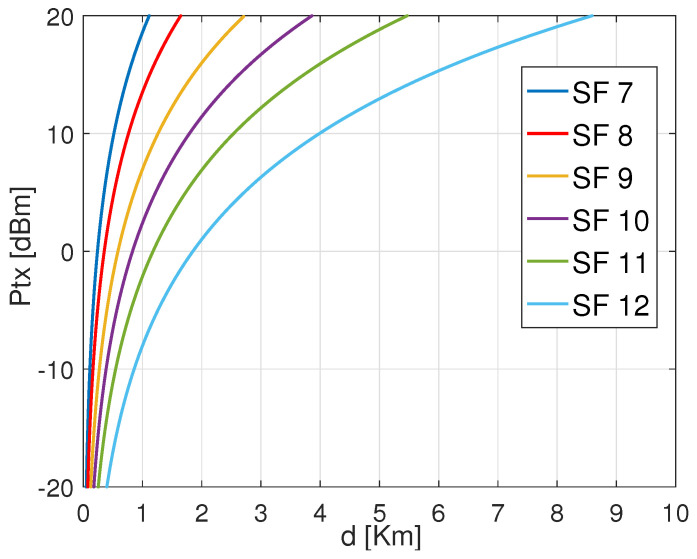
Transmission power $P_{tx}^{}$ versus range *d* for different SFs with CR 4/7 to carry out 30 Bytes payload.

Figure 3 shows the required transmission power of an ED using LoRa default ADR for different settings based on CR 4/7 and variable SFs from 7 to 12 when transmitting a payload of 30 bytes from variable distances. As shown, SF 12 requires lower transmission energy to attain the same distance, followed by SF 11, SF 10, SF 9, SF 8, and SF 7. Besides, SF 12 can attain the most significant distance with minimum energy dissipation. This is explained by the significance of CSS modulation to gain power in order to target long-range distances. Furthermore, from Figure 3, we state that for each specified combination between SF and CR has a threshold distance that can be covered according to the adopted transmission power by the ED.

**Figure 4:**
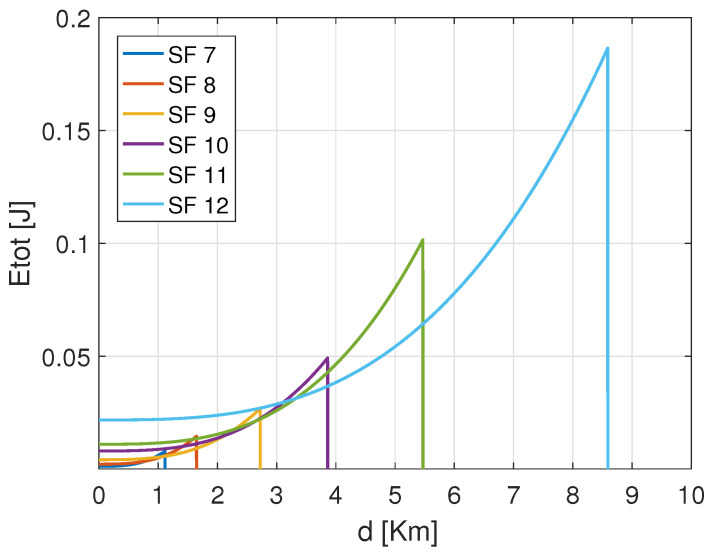
Total consumed energy versus distance *d* using SF 7 with CR 4/7 to carry out 30 Bytes payload.

Figure 4 shows the total consumed energy of an ED that transmits the same payload from distinct distances to the GW using the configurations based on CR 4/7 with variable SFs. The exhibited results confirm that the energy consumption increases with the increase of both the distance and the SFs. Among the SF configurations, SF 7 reveals to consume less energy than the others in short covered distances. Besides, from Figure 4, it is important to notice that for the same CR 4/7. Before SF 7 reaches its maximum critical distance, which is approximated to 1.2 Km, there is an interval where SF 8 consumes less energy than SF 7. This is noticed for the rest of the configurations. That signifies that if the energy consumption increases, there are some distance intervals where the next SF (i.e., SF 8) is better than the current one (i.e., SF 7). For instance, the curve representing the performance of the setting (SF 10, CR 4/7) was revealed to consume more energy than the following settings (i.e., (SF 11, 4/7) and (SF 12, CR 4/7)) even though it does not yet reach the maximum allocated transmission power $P_{tx}^{}$. Therefore, this declines the assumption that the ED should operate with a given setting until reaching the maximum $P_{tx}^{}$ before switching to the next configuration. The reason is that there is a trade-off between the processing and transmission energy for every single configuration, which approves our assumption about the energy performance in LoRa EDs. Hence, it is essential to notice that each SF configuration ensures energy efficiency on specific distance margins. That means the SF should be used for the distances where it may consume minimum energy to maintain energy efficiency.

**Figure 5:**
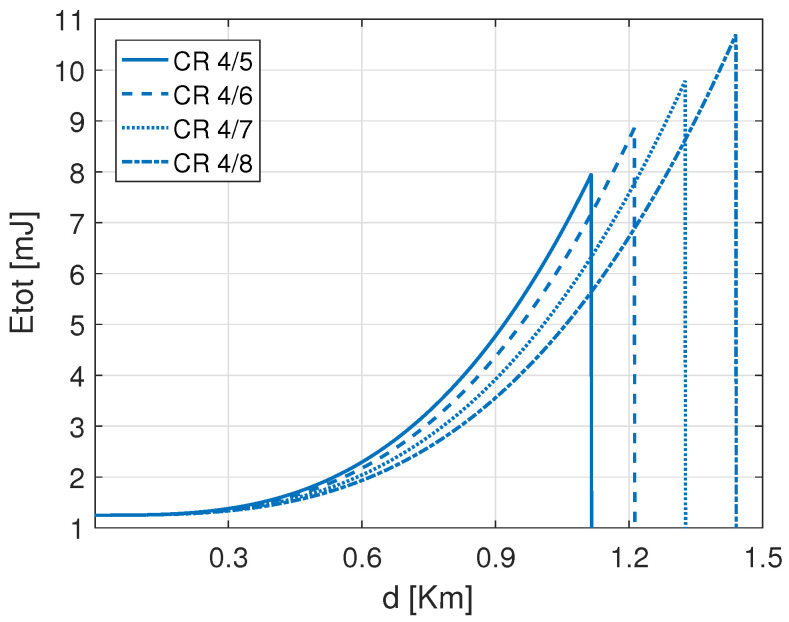
Total consumed energy at different ranges *d* using different CRs with SF 7 to carry out a 30 Bytes payload.

Figure 5 shows the total consumed energy of the configuration SF 7 using different CRs of LoRa default ADR. For the same SF 7, using CR 4/5 consumes more energy than CR 4/6, followed by CR 4/7 and CR 4/8. Moreover, the maximum distance that can be reached by CR 4/5 is shorter than the one reached by CR 4/6, CR 4/7, and CR 4/8, respectively. Therefore, CR 4/8 presents an advantage as it extends the covered distance with less energy consumption. That is justified by the BER gain performance of lower CR, which is better as it benefits from extra redundancy bits. Besides, according to the covered distance, the energy-efficient coding rate can be used to lower the ED energy consumption. In terms of energy-saving, the lower CR (i.e., CR 4/5) shows better performances toward time-on-air (TAO) since it is the fastest option. However, Hamming’s coding scheme, with its various CRs, has been selected to be used in the LoRa standard due to its lightweight coding, decoding, and parity check redundancies, which do not impact the performance intensively toward time-on-air. Accordingly, from both Figure 4 and Figure 5, it is inferred that both coding and modulation gains affect the energy consumption of LoRa EDs. Therefore, adopting the better setting configuration between SFs and CRs is of paramount importance.

**Figure 6:**
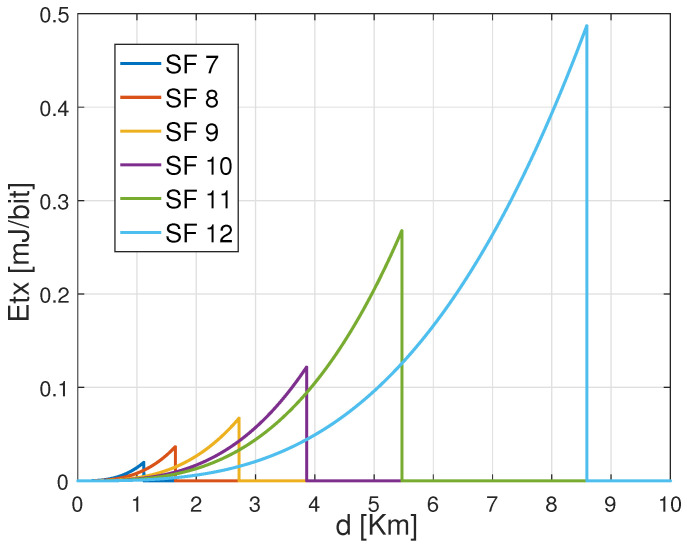
The transmission power performance relative to the range in Km carrying out a PL = 30 Bytes using setting of different SFs with CR 4/7.

**Figure 7:**
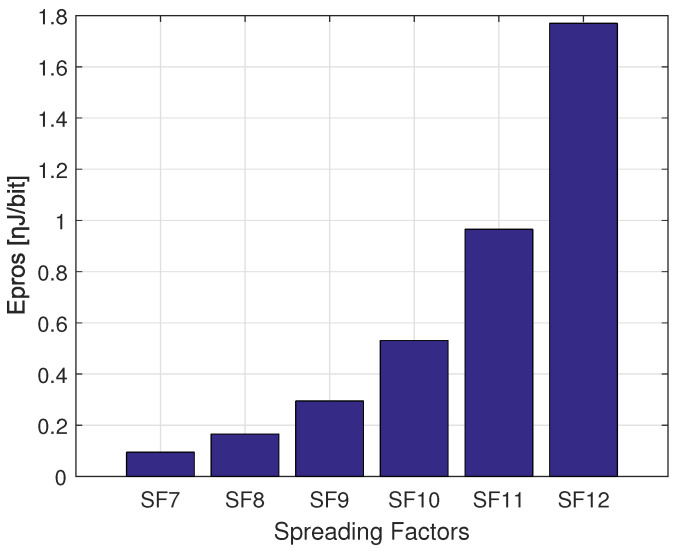
The distribution of the consumed energy for processing when transmitting a PL = 30 Bytes using different SFs setting with CR 4/7.

**Figure 8:**
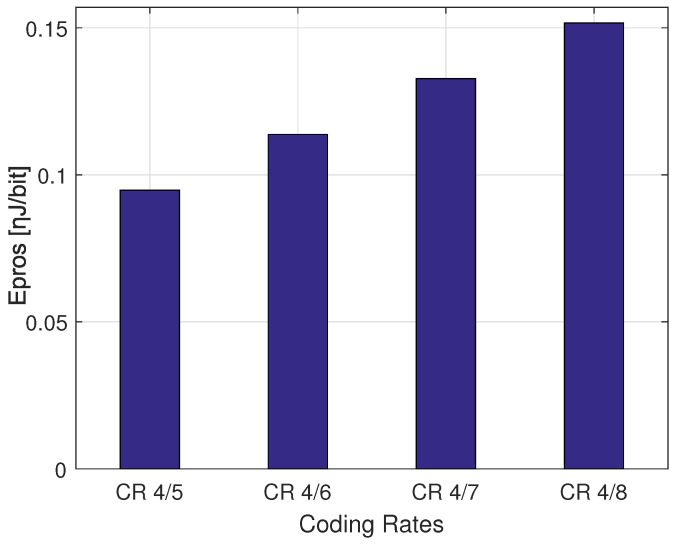
The distribution of the consumed energy for processing when transmitting a PL = 30 Bytes using setting SF 7 with different CRs.

The difference in the consumed energy for transmission and processing trade-offs can be derived respectively from Figure 6, Figure 7 and Figure 8. As shown, the smaller SF consumes more energy for transmissions $E_{tx}^{}$ operations and less energy for processing $E_{pros}^{}$, whereas the higher CR (i.e., CR 4/5) consumes less energy for transmitting the same payload of 30 Bytes using SF 7. More precisely, Figure 6 shows that SF 7 consumes more energy for transmission covering the distances inferior to 1.1 Km, and SF 8 consumes more energy than SF 9, SF 10, SF 11 and SF 12 when covering the distances between 1.1 Km and 1.8 Km. According to Figure 7 the approximated consumed energies for processing processes $E_{pros}^{}$ are 1.78 ηJ/bit, 0.97 ηJ/bit, 0.57 ηJ/bit, 0.31 ηJ/bit, 0.18 ηJ/bit and 0.11 ηJ/bit for SF 12, SF 11, SF 10, SF 9, SF 8 and SF 7 respectively when using same CR 4/7. Besides, Figure 8 shows 0.16 ηJ/bit, 0.14 ηJ/bit, 0.12 ηJ/bit and 0.09 ηJ/bit for the coding rates 4/8, 4/7, 4/6 and 4/5, respectively when using same SF 7.

Accordingly, the small SF consumes less energy in short distances than higher SFs. For this reason, the default LoRa mechanism configures the setting beginning from the smallest SF (i.e., SF 7) until reaching the highest available $P_{tx}^{}$ to access the maximum distance. Then the system switches to the immediate next SF ( i.e., SF 8) until the maximum SF 12. Nevertheless, this strategy is not always appropriate. As previously shown by Figure 4, it is revealed that there are distance ranges where the next SF (i.e., SF 8) configuration is better than the current SF (i.e., SF 7) in terms of energy consumption and reliability considerations. More specifically, it is not obvious to wait until achieving the maximum allowable $P_{tx}^{}$ to switch to the next setting; this applies to both SF and CR parameters allocation. For this reason, the proposed scheme aimed to address this gap to assign accurately the transmission parameters configuration for LoRa EDs.

**Figure 9:**
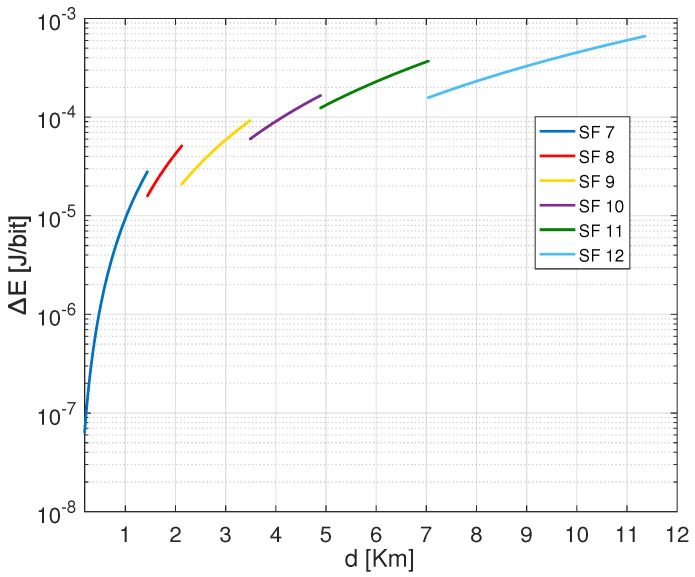
Optimum setting classifications for ranges *d* using different SFs with CR 4/8 to carry out 30 Bytes payload.

Figure 9 shows the transmission parameter’s energy classification for the standard LoRa/LoRaWAN scheme in function of distances. As illustrated, each configuration of SF and CR can guarantee an energy-efficient transmission in a specific range area defined by critical distances. The covered distance by a given parameter is controlled by the $P_{tx}^{}$ threshold. For example, when transmitting a PL of 30 Bytes using CR 4/8 from the distances 1 m ≤d≤1440 m; SF 7 appears to be the most efficient setting among others. whereas, SF 8, SF 9, SF 10, SF 11 and SF 12 show better performance respectively in 1441 m ≤d≤2127 m, 2128 m ≤d≤3489 m, 3490 m ≤d≤4892 m, 4893 m ≤d≤7041 m, and 7042 m ≤ d ≤ 11,360 m. Noting that these threshold distances may change by changing the coding rate or the payload length. Furthermore, the configuration assigned to cover the ranges should differ if the path loss exponent α differs.

**Figure 10:**
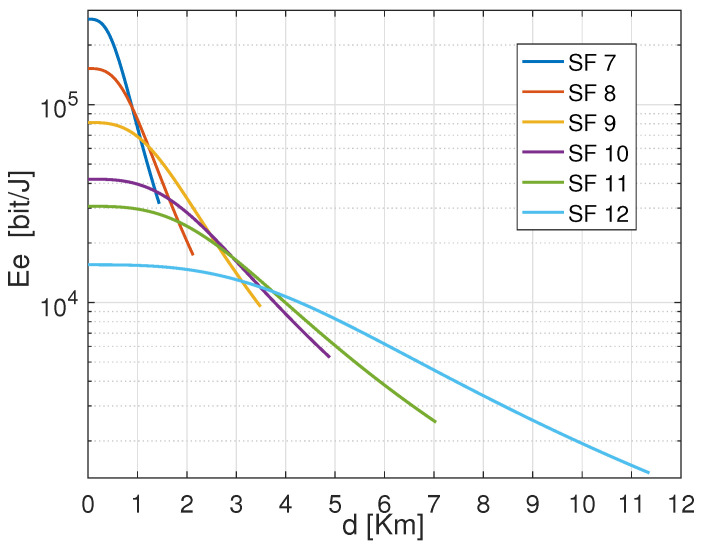
Energy efficiency distribution relative to variable covered distance *d* using different SFs with CR 4/8 to carry out a 30 Bytes payload.

Figure 10 illustrates the energy efficiency performance of LoRa default ADR using different SFs versus the covered distances. It is observed that the energy efficiency varies for different SFs and declines regardless the covered range. SF 7 reveals to be more efficient for short distances than others. Besides, each parameter setting shows its effectiveness within a specific range. However, the ranges classification associated to each SF shown in Figure 9 for LoRa default ADR could be updated if the energy efficiency performance for each tuning is considered. The most considerable configuration should prove the highest energy efficiency performance. Thus, if the energy efficiency of each setting is considered, the setting that would be used to cover variable ranges are respectively; (SF 7, CR 4/7) for the EDs situated at ranges below 855 m, (SF 8, CR 4/8), (SF 9, CR 4/8), (SF 10, CR 4/8), (SF 11, CR 4/8) and (SF 12, CR 4/8) for ranges 856 m ≤d≤1230 m, 1231 m ≤d≤2456 m, 2457 m ≤d≤3005 m, 3006 m ≤d≤3723 m and 3724 m ≤ d ≤ 11,360 m respectively.

In short, the difference in energy efficiency explains why several distances are considered thresholds at which specific parameters cannot succeed in communication. Therefore, the ED would determine the parameter autonomously to be considered at a defined distance to make a reliable transmission. Bearing this in mind, in other cases, the transmission can be successfully maintained using different options, even though the energy efficiency of any transmission would be taken into account to ensure a high level of energy management satisfactory. To this end, the critical distance $d_{c}^{}$ has the potential to increase the reliability of the overall communication setup and can also lead to conscious energy resource management.

**Figure 11:**
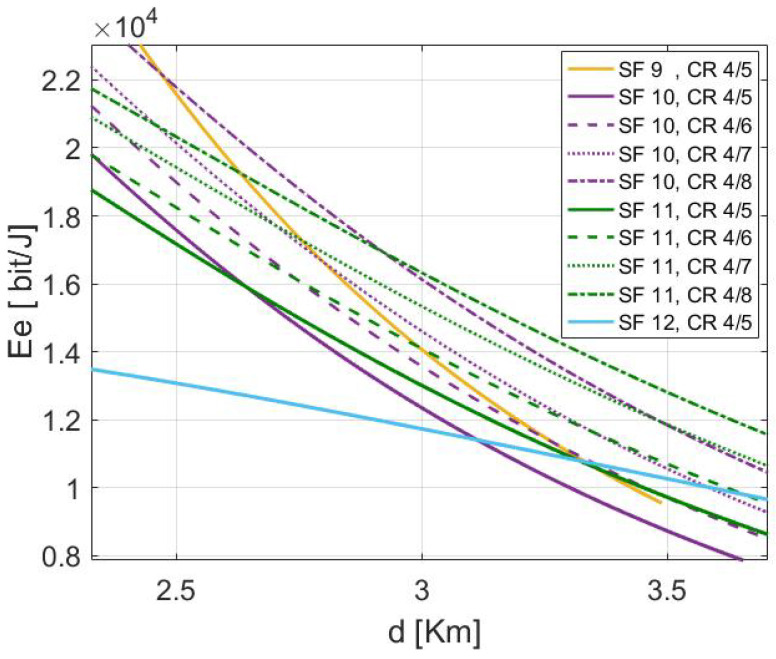
Energy efficiency performance comparison between of different possible transmission settings at the distance *d* = 3 km.

**Figure 12:**
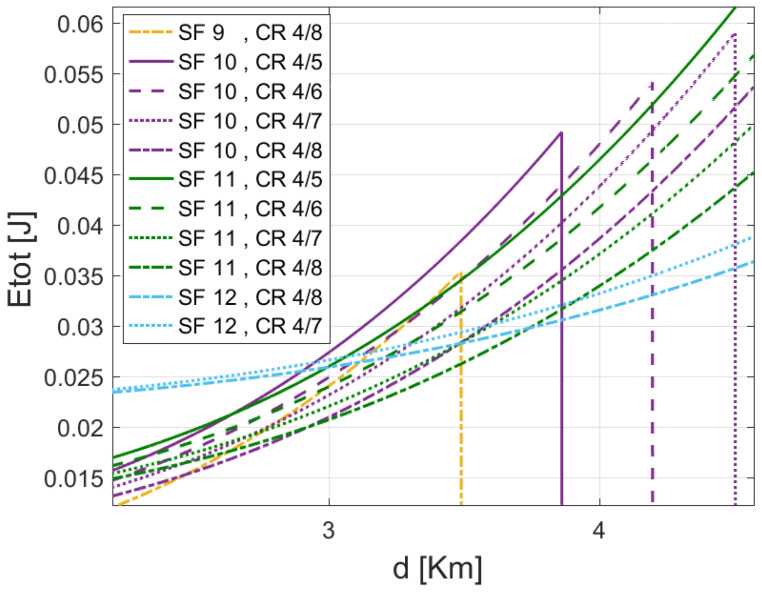
The total consumed energy by different possible transmission settings at the distance *d* = 3 km.

As exhibited by Figure 10, it is impossible to ED to succeed in transmission from it is position using the transmission settings (SF 7, CR 4/8), and (SF 8, CR 4/8). Nevertheless, the ED can reach the required distance by relying on four possible configurations based on SF 9, SF 10, SF 11, and SF 12 for the same CR = 4/8. However, the setting offering a considerable energy efficiency requirement is the most recommended. According to classical LoRa functioning, the ED would use the possibility (SF 9, CR 4/8) since the position of the ED is included in the covered ranges by this setting and the $P_{tx}^{}$ did not yet reach its threshold, which is associated with the maximum distance 3.4 km. This is explained by the fact that in the classical LoRa ADR, the system does not switch to the next SF or CR until the outflow capacity of the actual parameter exceeds the maximum $P_{tx}^{}$ threshold (i.e., the critical distance $d_{c}^{}$). However, according to Figure 11 and Figure 12, this configuration is not the most efficient when considering the energy efficiency and the total consumed energy of the possible setting that can succeed in the transmission of the ED.

### 6.2. Performance Evaluation of the Proposed Scheme versus LoRa Default ADR

In this section, we investigate the MDP based approach’s performance against the LoRa standard ADR for the same scenario conditions. For first scenario, we pick a random ED from the network that is situated at a distance of d = 3 Km from the GW. The network is supposed to cover an urban area. The concerned ED can operate dynamically with maximum transmission power up to +20 dBm and is instructed to handle the transmission parameters to convey payloads with different payload sizes. The comparison relies on the gains in transmission reliability and the energy consumption layouts. Initially, we study all the possible transmission parameters that can carry the transmission from the required distance. For this reason, we plot the curves of different possible combinations in the following figures to assess their performance in terms of energy consumption. Then, we compare the performance of the parameter assigned by LoRa default ADR versus the proposed scheme.

**Figure 13:**
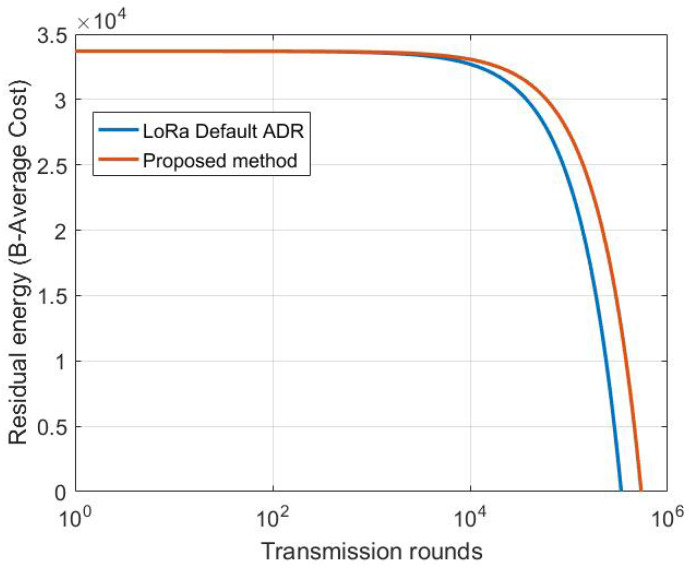
Residual energy variation versus distance of MDP-based model and LoRa default ADR carrying out a 30 Bytes payload.

Figure 13 shows the difference of energy residual distribution of the ED situated at 4 km from the GW using MDP-based method and default LoRa ADR. From this figure we conclude that our proposed model consumes less energy for the same scenario, as it helps the ED to carry on more transmission before it dies. MDP-based carry out 540,256 transmissions, 55,489 more than LoRa ADR. This is explained by the fact that our model adopts the parameter selection according to the channel condition taking into account both the transmission and processing energy of each possible setting before its selection.

**Figure 14:**
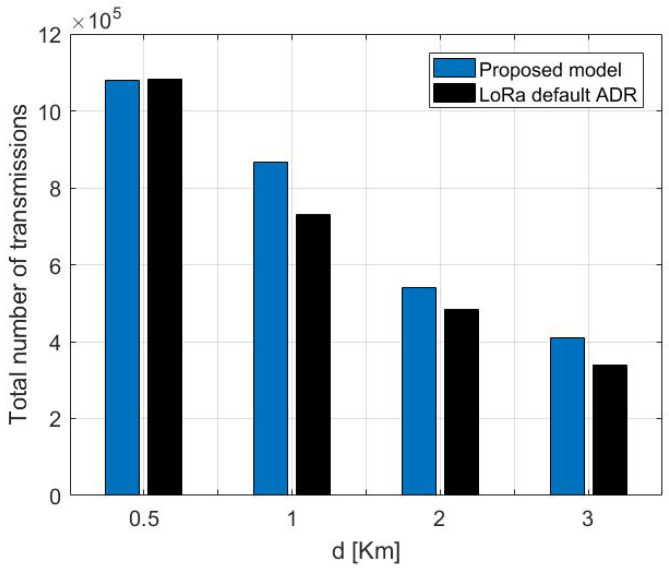
Total number of succeed transmission of the proposed model against LoRa default ADR for distinct distances transmitting a payload of a 30 Bytes.

To emphasize the results, we have simulated our model against LoRa default ADR under different scenarios for transmitting the payload size from distinct locations on the network. Figure 14 shows the results of the total succeeded transmission from 5 km, 1 km, 2 km, and 3 km, respectively, of the proposed model against LoRa ADR. As shown in Figure 14, the proposed model outperforms the standard LoRa ADR mechanism, leading the ED to carry out more transmissions before dying. For instance, from the distance of 1 km, the ED that adopts default LoRa ADR 867,565 maximum transmissions, which is less than 135,826, is achieved by our proposed model. Concerning the ED situated at 2 km, it achieved 540,256 transmissions with 55,489 more than LoRa defaults ADR. As noticed, the number of total transmissions that the ED can realize decreases with increasing the distance, which signifies that the ED that is situated far from the GW is expected to die before the nearest ones. Additionally, the energy consumption increases as the payload size increases.

**Figure 15:**
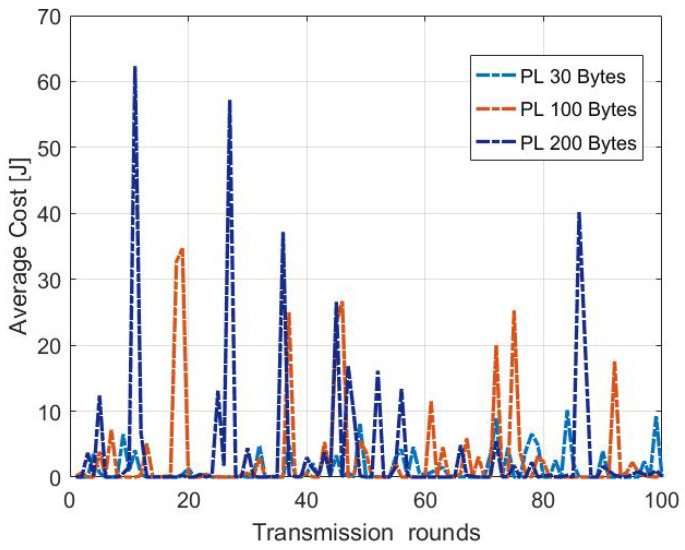
Average cost variation of each round for different package sizes from 3 km using MDP-based model.

Figure 15 shows the average energy cost distribution by the 100 first transmission rounds using MDP model. The total consumed energy $E_{tot}^{}$ differs from one round to another due to the random channel conditions. Our proposed model adopts the transmission parameters according to the channel medium. Therefore, the average cost changes from a round to another due to the randomness of the channel medium. Besides, the cost increases with increasing the payload size. The larger the payload size, the more energy the ED is expected to consume, therefore, it will last for less time.

**Figure 16:**
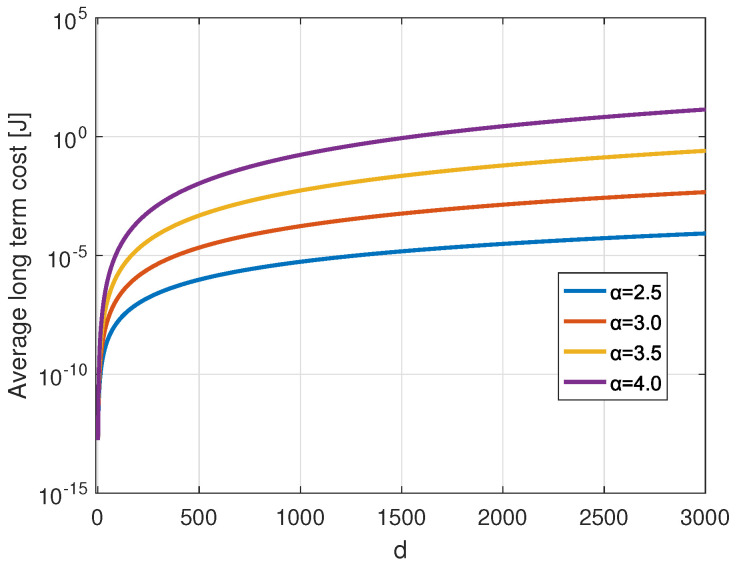
The average long term cost of transmitting a PL = 30 Bytes using MDP method under different channel states versus distances.

Figure 16 presents the average long-term cost against the distance variation for transmitting 30 Bytes payload for different channel states *H*. For channel states that have been selected for the study; α = 2.5, 3, 3.5 or 4. From the figure, we conclude that the MDP average cost increases with the transmission distance and the path loss exponent representing the channel condition, including the shadowing. Our model for each distance chooses the set of parameter selections that consumes minimum energy. For each channel state, our model succeeds in choosing the better policies that reside on choosing the adequate SF and CR for a given transmission, whatever the position of the ED on the network.

The total number of transmissions that an ED can undergo per day in public LoRa networks is limited due to the region and state restrictions. The DC obeys the ISM frequency band; thus, the maximum duty cycle allowed is 1%. After completing a given transmission, the ED must remain silent for 99% of the total time taken. More precisely, the ED forwards a packet within a time interval and then waits for a given time as instructed by the DC duration. Besides, to predict the lifespan and the total number of transmissions that can be held by an ED equipped with different battery source capacities (500 mAh, 2600 mAh, and 3500 mAh) that transmit every DC, the same payload packet of 30 Bytes using the same settings. We apply the experience scenarios of different battery capacities to the identical previous ED. Then we compare its lifespan when adopting the proposed method versus the default LoRa based ADR.

**Figure 17:**
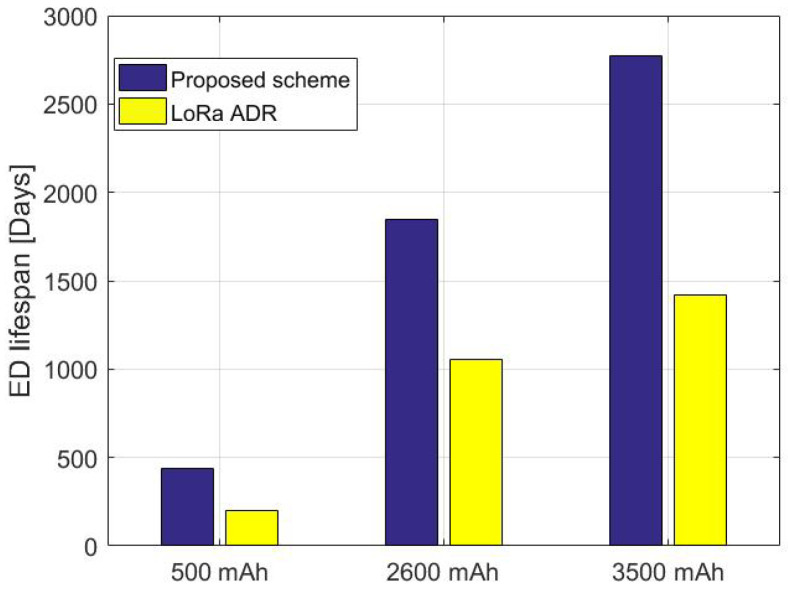
Comparison of the proposed scheme against LoRa’s default ADR in terms of ED lifetime for different battery capacity.

Figure 17 shows the performance of the studied ED relying on our method as well as on the LoRa defined scheme assuming a DC 1%. The ED’s lifespan depends on the used batteries’ capacity for both methods. In these scenarios, adopting the configuration recommended by our proposed method, the ED lasts for more time than using LoRa default ADR. The ED can last for approximately 435 days, 1850 days, and 2771 days using a battery capacity of 500 mAh, 2600 mAh, and 3500 mAh, respectively. Meanwhile, it may last less time by adopting LoRa ADR for the same battery capacities; 202 days, 1052 days, and 1417 days. As a result, the proposed scheme is expected to increase the times more than the LoRa standard ADR. Accordingly, the lifespan of the ED may last for more when transmitting payloads of size smaller than PL = 30 Bytes, and it may be decreased by increasing the length of the transmitted payload.

## 7. Conclusions

Several works are interested in using the technology without deeply launching its functional mechanisms. Likewise, these networks have become denser with the increased number of connected devices on the network. Therefore, evaluating the overall network performance, such as by measuring the lifespan, tracking the energy consumption, and evaluating its reliability and scalability, becomes more demanding. This paper highlights the IoT paradigm’s energy efficiency and reliability performance of LoRa EDs. Firstly, it provides comprehensive knowledge about the functioning of LoRa transceiver operations, focusing on the CSS modulation and the channel encoding schemes. Secondly, it provides a thorough mathematical model devoted to assessing the energy consumption in LoRa EDs. The model concentrates more on communication and processing energy, which has not been considered in most previous works. Accordingly, this model could assist in forecasting the LoRa networks’ lifespan in general or anticipating the lifespan of a single ED on the network regarding its distance from the GW before the real implementation of the network. Thirdly, it presents an MDP-based energy-efficient adaptive scheme that serves to tune LoRa physical transmission parameters. More precisely, it permits LoRa EDs to tune their radio transmission parameters, ensuring energy efficiency and the reliability of packet delivery. The scheme compares and evaluates the processing and transmission energy trade-off of each possible setting of any selection. The process aims to assign an accurate set of parameters regarding the BW, CRs, SF, transmission power, payload size, and the position of the loRa EDs. This approach has been evaluated and compared with LoRa default ADR. The obtained results through simulations have shown that the proposed method provides a better performance than LoRa default ADR in energy consumption in the studied scenarios, which leads to an increase of the LoRa end-nodes lifespan. Additionally, the presented energy model accurately anticipates the node’s lifespan for different transmission scenarios regarding the payloads’ size and duty cycle periods. In future works, we aim to adapt our proposed scheme to address the energy efficiency and reliability for dynamic EDs where the adaptive physical layer parameter management is complex and challenging. Moreover, the future research will deal with multi-objective optimization in mobile edge computing. We plan to design architecture based on Mobile Edge Computing between the Gateways and the connected objects. All the processing charges of the IoT devices, whatever the nature (Objects, Drones, Vehicles, Robots, and more), will be sent to the Edge Server, in which a multi-objective optimization will be run to select the transmission and processing parameters of the whole network, then forward the optimal decisions via a downlink transmission. We believe this approach will provide more energy and delay the performance of the IoT networks.

## Figures and Tables

**Table 1:** Simulation parameter values.

Parameter	Value
BW	125 KHz
CR	4/5, 4/6, 4/7, 4/8
SF	7, 8, 9, 10, 11, 12
ED type	Class A
Path loss exponent	2.5≤α≤4
$N_{F}^{}$	10 dBi
DC	1 %
Sensing energy per cycle	0.28 mJ
$T_{sleep}^{}$ + $T_{wakeup}^{}$	0.18 μs
$I_{leakage}^{}$	10 ηA
$I_{rx}^{}$	11 mA
$I_{sleep}^{}$	1.5 μA
$I_{pros}^{}$	22 mA
Processing frequency	4 MHz
Carrier frequency	868 MHz
Sensor unit voltage	2 V
Leakage voltage	2.2 V
Processing unit voltage	3.3 V

## Data Availability

Not applicable.

## Abbreviations

The following abbreviations are used in this manuscript:

**Acronym**	**Description**
ADR	Adaptive Data Rate
BER	Bit Error Rate
BW	Bandwidth
CR	coding Rate
CPU	Central Processing Unit
CSS	Chirp Spread Spectrum
*d*	distance
$d_{c}^{}$	critical distance
DC	Duty Cycle
ED	End devices
Ee	Energy Efficiency
$E_{c}^{tx}$	Uncoded Transmission Energy per bit
$E_{Tot}^{}$	Total consumed Energy per cycle
$E_{act}^{}$	Consumed Energy in sleep mode
$E_{tx}^{}$	Consumed Energy for transmission
$E_{rx}^{}$	Consumed Energy during reception
$E_{rx,w1}^{}$	Consumed Energy during W1 opening
$E_{rx,w2}^{}$	Consumed Energy during W2 opening
$E_{proc}^{}$	Processing Energy
$E_{c}^{}$	Circuity Energy
$E_{s}^{}$	Sensing Energy
$E_{Network}^{}$	Total consumed energy of the network per round
$E_{Leakage}^{}$	Consumed energy for leakage per round
FEC	Forward Error Correction
$f_{cpu}^{}$	Processing Frequency
GW	Gateway
ISM	Industrial Scientific and Medical
IoT	Internet of Things
LoRa	Long Range
LPWAN	Low Power Wide Area Network
MAC	Medium Access Control
$M_{T}^{}$	Modulation time
$M_{speed}^{}$	Modulation speed
$N_{F}^{}$	Noise Figure
PER	Packet Error Rate
PHY	Physical Layer
Ptx	Transmission Power
SF	Spreading Factor
SNR	Signal To Noise Ratio
$S_{rx}^{}$	Receiver sensitivity
$T_{c}^{}$	Chirp duration
$T_{s}^{}$	Symbol duration
$T_{w1}^{}$	First Window listening duration
$T_{w2}^{}$	Second Window listening duration
